# Population Adherence to Infection Control Behaviors during Hong Kong’s First and Third COVID-19 Waves: A Serial Cross-Sectional Study

**DOI:** 10.3390/ijerph182111176

**Published:** 2021-10-24

**Authors:** Emily Ying Yang Chan, Jean H. Kim, Kin-on Kwok, Zhe Huang, Kevin Kei Ching Hung, Eliza Lai Yi Wong, Eric Kam Pui Lee, Samuel Yeung Shan Wong

**Affiliations:** 1 Collaborating Centre for Oxford University and CUHK for Disaster and Medical Humanitarian Response (CCOUC), The Chinese University of Hong Kong, Hong Kong, China; huangzhe@cuhk.edu.hk (Z.H.); kevin.hung@cuhk.edu.hk (K.K.C.H.); 2 GX Foundation, Hong Kong, China; 3 JC School of Public Health and Primary Care, The Chinese University of Hong Kong, Hong Kong, China; JHKim@cuhk.edu.hk (J.H.K.); kkokwok@cuhk.edu.hk (K.-o.K.); lywong@cuhk.edu.hk (E.L.Y.W.); lkp032@cuhk.edu.hk (E.K.P.L.); yeungshanwong@cuhk.edu.hk (S.Y.S.W.); 4 Accident & Emergency Medicine Academic Unit, The Chinese University of Hong Kong, Prince of Wales Hospital, Hong Kong, China

**Keywords:** COVID-19, urban, health risks, serial cross-sectional, pandemic, population adherence, infection control behaviors, Hong Kong

## Abstract

Background: Although COVID-19 has affected over 220 countries by October 2021, there is limited research examining the patterns and determinants of adherence to infection control measures over time. Aims: Our study examines the sociodemographic factors associated with changes in the frequency of adherence to personal hygiene and social distancing behaviors in Hong Kong. Methods: A serial cross-sectional telephone survey in the general population was conducted during the first (March 2020) (*n* = 765) and third wave (December 2020) (*n* = 651) of the local outbreak of the COVID-19 pandemic. Respondents were asked about their level of compliance with various personal hygiene and social distancing recommendations. Results: By the third wave, mask use increased to 100%, and throughout the study periods, >90% practiced frequent hand hygiene. However, adherence to social distancing measures significantly waned over time: avoidance of social gatherings (80.5% to 72.0%), avoidance of public places/public transport (53.3% to 26.0%), avoidance of international travel (85.8% to 76.6%) (*p* < 0.05). The practice of ordering food takeout/home delivery, however, increased, particularly among high-income respondents. Higher education, female gender and employment status were the most consistently associated factors with adherence to COVID-19 preventive practices in the multivariable models. Conclusions: In urban areas of this region, interventions to improve personal hygiene in a prolonged pandemic should target males and those with low education. In addition to these groups, the working population needs to be targeted in order to improve adherence to social distancing guidelines.

## 1. Introduction

The outbreak of novel coronavirus 2019 (COVID-19) has been a significant global public health threat since its emergence in December 2019. As of 3 October 2021, the SARS-CoV-2 virus has spread to over 220 countries with 234 million reported cases and 44 million deaths [1]. Although the novel coronavirus has shown high transmissibility and high case fatality rates in many regions of the world, national responses to COVID-19 have varied greatly among the affected countries [2,3]. During the early phase of the pandemic, lockdown orders were made to restrict the movement of citizens in many countries across Europe and Asia [4,5,6]. By contrast, countries such as Sweden and Iceland did not close schools and have allowed citizens to freely leave their homes throughout the pandemic [7,8,9,10].

Hong Kong is a large regional travel hub in southern China with 7.5 million residents, and each person has a mean daily number of 12.5 social encounters of with people [11]. Similar to many regions of the world, Hong Kong experienced successive waves of COVID-19 outbreaks since late January 2020 [12]. Social distancing measures such as avoidance of public gatherings, avoidance of restaurant dine-in services, minimizing use of public transport, and avoidance of travel to high COVID-19-risk regions were considered to be effective interventions in reducing the spread of the pandemic [13,14]. Moreover, uptake of recommended personal hygiene behaviors such as wearing masks and frequent handwashing is a key factor in the control of this highly transmissible virus. Bringing one’s own eating utensils when dining out and the use of serving utensils for shared dishes have also been shown to be effective in reducing transmission [15,16,17,18,19,20,21,22]. To control the local outbreak, the Hong Kong health authorities and public health experts (a) promptly implemented a series of non-pharmaceutical measures including school closure, work from home arrangement for civil servants, reduced restaurant hours and closure of bars [23]; (b) actively recommended mask use and frequent hand sanitation very early in the pandemic [24,25]. The level of adherence of these measures is of public health interest since it relies on temporal changes in population behaviors, which were shaped by individuals’ ability to perceive risks associated with the virus and to adapt their behaviors accordingly.

A number of studies conducted in the U.S., Europe and Africa had observed a gradual decline in adherence to COVID-19 preventive measures as the pandemic evolved [26,27,28,29]. However, other studies have noted that adherence to hygiene measures and social distancing recommendations have remained high during this pandemic [30,31] and that the intention to adhere to protective measures was also high [32,33]. The determinants that induced adherence and compliance to these measures were unclear. Previous studies have demonstrated that adoption of the protective behaviors is associated with various sociodemographic characteristics [31,32,33,34,35,36,37,38,39,40,41]. In particular, women and those with higher education were noted to have greater compliance with health protection behaviors [34,41]. It may be that women, who are more likely to be primary caregivers to their children in the household may be more cautious of the infectious diseases [36], while those with higher education may have better knowledge of the risk factors and the efficacy of the preventive measures in the community spread of infections. Since health literacy is also correlated with income levels and working status is likely to be associated with risk perceptions during pandemics, these factors are also likely to influence compliance with and maintenance of infection control behaviors [34,35]. Moreover, being employed may incur higher risk of infection since these people are more likely to commute to work and more likely to have interpersonal contact with people outside the home. The work culture of some communities may also make it difficult to maintain social distancing [36]. Lastly, older individuals and those with pre-existing health conditions that put them at risk of greater adverse sequelae of COVID-19 infection such as hospitalization and death are likely to have greater concern about infection and presumably would practice more preventative behaviors. 

Even though the effect of demographic, geographic and psychological characteristics for the uptake of protective behaviors has been studied extensively during pandemics, the interaction between these factors and the role of further mediators are not yet clear [41]. Furthermore, non-adherence to COVID-19 regulations may be due to structural vulnerability rather than beliefs or fatigue [32,33]. These factors have not been examined comprehensively in a setting, such as Hong Kong, that has experienced successive waves of pandemics. In order to allow rapid, targeted interventions to the appropriate risk groups, it is, therefore, important to be able to identify risk groups for non-adherence to recommended guidelines. 

Hong Kong was the epicenter of several pandemics in the last two decades. Since 1997, Hong Kong has experienced several waves of human cases of avian influenza including H5N1 and H7N9 [13,17]. More significantly, the severe acute respiratory syndrome (SARS) pandemic was first identified in Hong Kong in 2003 [42,43]. During the SARS epidemic, Hong Kong did not undergo a lockdown despite 1755 local cases, but it was noted that the community widely adopted various preventive behaviors. Mask use rose and increased hand hygiene rose to approximately 95% of the population, while the avoidance of crowded places and home disinfection rose to over 80% of the population [16]. By contrast, a study conducted between 2005 and 2008 of H5N1 in which no human deaths occurred in Hong Kong, found that the levels of preventive behaviors that the public anticipated adopting in the event of human-to-human transmission declined (73.8% to 43.7% for mask use and 72.6% to 51.5% for avoidance of public places) [17]. There is, however, limited understanding of the pattern and how Health-Emergency Disaster Risk Management (Health-EDRM) personal preventive behaviors may evolve with the prolonged biological emergency.

Enactment of public health and social measures including physical and social distancing measures (e.g., lockdowns and restaurant/bar restrictions) and personal measures (e.g., hand hygiene/mask wearing) was shown to contribute to halt the spread of the disease [44]. In light of this, we aimed to examine the population adherence to these infection control behaviors in a region whose population is highly experienced in pandemic response due to a long history of zoonotic epidemics. It is theorized that due to Hong Kong’s past experience with successive pandemics [45], the population would rapidly adopt high levels of personal hygiene (e.g., mask wearing) and social distancing guidelines in the first local wave. The study also will examine changes from the first wave of the pandemic when only 766 local cases and 5 deaths were confirmed in Hong Kong with the third wave of the pandemic when the total confirmed local cases had risen to 8725 with 179 deaths. It is theorized that the personal hygiene and social distancing behaviors would increase during the third wave due to higher perceived susceptibility (from much higher daily caseloads) and perceived severity of COVID-19 (from much higher numbers of deaths). In addition, the study aims to identify the sociodemographic risk groups for non-compliance with these preventive behaviors to allow for rapid interventions to these target groups. By shedding light on patterns of infection control compliance in a large metropolis with previous SARS and avian influenza pandemic experience, these findings may provide insights for post-COVID-19 pandemics in other regions of the world that have limited pandemic experience.

## 2. Materials and Methods

### 2.1. Study Design and Sampling

Serial cross-sectional telephone surveys were conducted using random digit dialing in Hong Kong at two time periods: (1) first wave of local epidemic (22 March to 1 April 2020) (*n* = 765) and (2) third wave of the local epidemic (*n* = 651) (15–29 December 2020). The sample sizes of 765 and 651 were accompanied with maximum margins of error of 3.5% and 3.8% at a 95% confidence level. The target population of the telephone survey were Cantonese-speaking Hong Kong residents aged 18 years or above, including individuals holding work or study visas. Stratified random sampling was used to ensure that the data collected were representative of the general population in Hong Kong in terms of district of residence. This data collection strategy has been used in similar local studies on infectious diseases [13,19]. Phone calls were made in the evening on weekdays and during the entire day on weekends to prevent overrepresentation of the unemployed. The household member with birthday closest to the interview date was selected for the baseline survey (“last birthday” method). After the purpose of the survey was explained and assurances of the confidentiality of response were made to potential respondents, oral consent was obtained from participants.

### 2.2. Data Collection and Data Management

Information about the respondent’s sociodemographic and background characteristics was obtained in both surveys as the main exposure variables. Specifically, the surveys obtained information about the respondent’s gender, age group, marital status (married versus all other), monthly household income, highest educational attainment, type of occupation and whether the individual had ever been diagnosed with a chronic health condition such as hypertension or diabetes (see Table 1). Since COVID-19 case distribution varied by geographic region, we also asked about the respondent’s area of residence (18 districts in Hong Kong). Respondents were also asked about the channels by which they obtained their COVID-19 news: television, internet, smartphone applications (Yes/No). 

For the outcome measures, respondents were asked about their compliance in adopting four personal hygiene measures (e.g., wearing a mask, handwashing) and four social distancing measures (e.g., avoidance of public transport) (see Table 2). For each protective measure, the level of adoption was assessed with four-point Likert scale responses (1 = always, 2 = usually, 3 = occasionally, 4 = never) during Wave 1 and Wave 3. Respondents were considered compliant with the preventive behavior if they responded that they “always” or “usually” engaged in the preventive behaviors, while those who responded that they only “occasionally” or “never” performed the behaviors were classified as non-compliant. These eight protective measures were used as outcome variables, while sociodemographic variables were used as predictors in the subsequent regression analyses.

### 2.3. Statistical Analysis

Proportion and frequency of responses were tabulated and shown in Table 1 and Table 2. Cohen’s w effect size was used to compare the demographics of respondents with the 2016 population by census in Hong Kong, while the chi-square test was used to examine population differences between Wave 1 and Wave 3 [46]. The four-point Likert scale responses for the preventive behaviors were dichotomized [47], whereby respondents were considered to be strong adherents if they responded 1: “Always” or 2: “Usually” to the item. Two-proportions z-test with continuity correction was used to test the difference of the personal protective measures between the two waves of studies. To examine the persistent sociodemographic factors independently associated with adherence with the personal hygiene and social distancing behaviors across time, we included all sociodemographic variables as candidate variables in the backward stepwise multiple logistic regression model, where the variable with the largest *p*-value was removed at each step. The multivariable models forced the time period (Wave 1 versus Wave 3) and age variable into the final model to adjust for the potential confounding effects. Adjusted odds ratio (AOR) in the logistic regression models with corresponding 95% confidence interval (95% CI) was used as a measure of independent association between an exposure and an outcome. Due to the 100% compliance with mask wearing in Wave 3, we did not further examine factors associated with compliance for this personal hygiene behavior. Separate analyses for the 1st and 3rd wave were conducted (see Appendix A) to examine factors associated with different protective measures adherence at two different study periods [48]. Statistical significance was set at α = 0.05. All analyses were performed using IBM SPSS 24 [49]. Ethical approval was received by the Survey and Behavioral Research Ethics Board of the university sponsoring the study.

## 3. Results

### 3.1. Characteristics of the Study Sample

**Figure 1:**
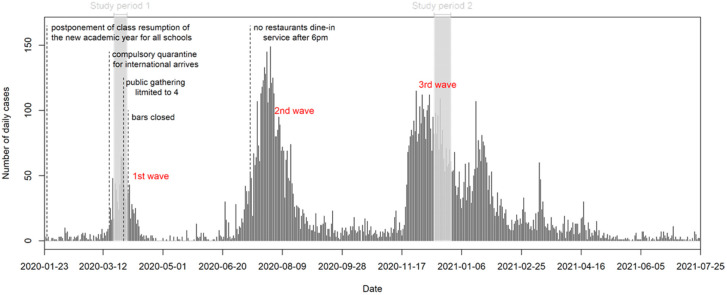
Hong Kong COVID-19 pandemic waves (2020–2021) with daily caseloads, government pandemic responses and timing of the serial cross-sectional studies.

A total of 765 and 651 participants were recruited in the baseline survey and second survey, respectively (see Figure 1). Between the two serial cross-sectional samples (Table 1), the respondents in the second survey sample were slightly older, slightly less educated and had lower income levels. Descriptive statistics of the personal characteristics of the study samples are shown with the comparison to the characteristics of the most recent Hong Kong population census. Although the study samples were comparable to the census characteristics, the third wave sample were older and had lower education status and household income than the first wave sample. In this study, none of respondents reported that they had been infected by COVID-19.

### 3.2. Compliance Levels with Recommended COVID-19 Preventive Behaviors across Time

Table 2 showed the practice of preventive measures against COVID-19 in the first and third epidemic wave. Overall, the self-reported practice of most of the hygiene preventive measures listed were high except for the practice of bringing one’s dining utensils when dining out. The cultural habit of bringing one’s own dining utensils (e.g., bringing personal chopsticks) to restaurants, which is occasionally practiced by the elderly or very young children, was not commonly practiced during this epidemic, with only 1 in 12 respondents reporting to have done this in the early part of the pandemic, and these percentages dropped further in the later stages. Wearing face masks, handwashing with soap, and strict use of serving utensils for shared dishes were practiced widely but only mask wearing showed a statistically significant difference between the first and third waves, rising to 100% of the surveyed respondents.

All four social distancing practices examined in this study showed statistically significant differences between the first and third waves of the study. Whereas respondents were significantly less likely to avoid international travel to high-risk regions, avoid social gatherings, avoid public places/public transport in the third wave, a much higher proportion of respondents adopted the use of food takeout/home delivery services to avoid eating in restaurants (Table 2).

### 3.3. Factors Associated with Compliance with Hygiene Measures against COVID-19

Table 3 shows the factors associated with the practice of various personal hygiene and social distancing measures after forcing the age variable and time period of the study (Wave 1 versus Wave 3). Due to the near ubiquitous use of face masks in Wave 1 and 100% compliance in Wave 3, we did not examine mask use further in these multivariable models. 

For personal hygiene behaviors, the results showed that while hand hygiene and the practice of bringing one’s own dining utensils did not show any significant change between Wave 1 and Wave 3, the strict use of serving utensils had declined by Wave 3. 

The practice of frequent handwashing was significantly higher among females, those with higher education (AOR for secondary education: 1.85, 95% CI: 1.07–3.20; AOR for post-secondary education: 2.03, 95% CI: 1.12–3.69) and non-elderly adults (AOR for aged 50–64: 1.61, 95% CI: 1.01–2.57; AOR for aged 35–49: 3.03, 95% CI: 1.62–5.69; AOR for aged 18–34: 3.19, 95% CI: 1.64–6.19). While adherence to the strict use of serving utensils at meals was significantly higher among females (AOR: 1.37, 95% CI: 1.06–1.78) and those with higher education (AOR for secondary education: 1.54, 95% CI: 1.01–2.35; AOR for post-secondary education: 1.88, 95% CI: 1.19–2.99). By contrast, younger-aged respondents (AOR: 0.41, 95% CI: 0.25–0.67), blue-collar workers (AOR: 0.50, 95% CI: 0.35–0.73), housewives (AOR: 0.61, 95% CI: 0.39–0.97) and non-employed individuals (AOR: 0.66, 95% CI: 0.44–1.00) were significantly less likely to practice this behavior as compared with elderly individuals and white-collar workers. The practice of bringing one’s own dining utensils to restaurants was only significantly lower among those between 50–64 years of age (AOR: 0.45, 95% CI: 0.22–0.92).

### 3.4. Factors Associated with Compliance with Social Distancing Measures against COVID-19

For the social distancing behaviors, the multivariable models revealed that the practice of all behaviors had significantly declined by Wave 3 except for the practice of ordering takeout/food delivery services, which had significantly increased during this time period. 

During the study periods, females, those with higher education and non-employed respondents were significantly more likely to avoid social gatherings (AOR: 1.44–2.65). Similarly, avoidance of public places/public transport was more likely to be practiced by those with higher education and non-employed respondents as well as housewives and full-time students (AOR: 1.61–3.53). Respondents under 50 years of age, higher-income individuals and males were more likely to use restaurant takeout/home delivery services (AOR: 2.33–2.45). For avoidance of international travel, only housewives were more likely to avoid traveling abroad (AOR: 1.85, 95% CI: 1.09–3.16) as compared with white-collar workers. Having a chronic health condition was not associated with any of the preventive behaviors examined in this study.

## 4. Discussion

Overall, study findings reveal that residents in Hong Kong, a city with a long history of worldwide pandemics, were quick to adopt basic COVID-19 preventive practices. In contrast to many other countries in which citizens have resisted mask wearing as an infringement of civil liberties, there was a rapid uptake of mask use among Hong Kong residents during the first wave of the pandemic when the caseload was still relatively low. In addition to the near ubiquitous uptake of mask wearing in the first local wave of the pandemic, there was a high degree of compliance with hand hygiene measures by the general population. Our results were consistent with previous findings from an avian influenza epidemic showing high adherence to mask use and lower adherence to social distancing measures [13]. Although mask wearing and hand hygiene did not significantly decline between epidemic Wave 1 and Wave 3, the majority of social distancing practices waned over time. This may be attributed to the decreasing perceived disease severity across time [50]. Even though nearly all respondents routinely checked the daily COVID-19 situation in Hong Kong, their adherence to social distancing significantly decreased during the much larger third wave of the local pandemic. These findings suggest that combatting pandemic fatigue in adhering to social distancing guidelines will be the major focus of ongoing government infection control efforts.

Our study identified sociodemographic determinants of preventive-behavior uptake in Hong Kong during the COVID-19 pandemic. In addition to older age and lower education level, male respondents were more likely to have poorer hand hygiene, which was consistent with studies conducted abroad [27]. Since the elderly are at higher risk of COVID-19 mortality, hand hygiene should be strongly promoted in this age group. Higher education status but not higher income was commonly associated with the adoption of multiple preventive behaviors. It is possible that those with higher education may be better at evaluating public health messages and integrating the recommended actions into their daily life. It is also possible that those with lower education have lower perception about the transmissibility of the disease, incorrect ideas about the transmission routes and inadequate knowledge of the potentially severe morbidity from COVID-19. This suggests that increased knowledge rather than financial resources are the main drivers of adoption of these preventive behaviors. Past studies conducted in during SARS and H5N1 noted that misconceptions were prevalent in the population with regard to transmission routes [15,16]. Hence, government public health messages, which have mainly served to encourage mask wearing, should also include information about the potential serious sequelae of non-fatal disease and emphasize the possible airborne and non-airborne transmission routes so that less educated individuals can have a better understanding of the risks. Moreover, work-related considerations are found to be important influences in the practice of these preventive behaviors. Housewives and non-employed individuals who have less need to leave the home were more likely to engage in social distancing measures such as avoidance of public places, public transport and social gatherings. Despite allowances for working at home by many industries in Hong Kong, a large proportion of workers in retail, hospitality and educational sectors were still required to come to work. Hence, protective measures in the workplace and in public transport need to be emphasized and maintained, particularly in high-density urban areas. 

As a likely result of intermittent prohibitions against evening dine-in services in Hong Kong restaurants since July 2020 [28,29,30], the use of food delivery/take out services greatly increased between the two study periods. Restaurant delivery services, which were rare in Hong Kong due to the close proximity of eating establishments to residential areas, quickly became available using mobile applications during this COVID-19 pandemic. It is unsurprising that younger people were more likely to use these services due to the digital nature of food ordering. Those from more affluent households were also more likely to use takeout/home delivery services since these services usually incur delivery costs or minimum spending requirements [31]. It is also likely that males used these food ordering services more frequently than females, who tend to prepare and consume homemade meals.

The Hong Kong government has recommended the strict use of serving utensils during communal meals since the SARS epidemic in 2003 since Chinese cuisine consists of communally shared dishes rather than individually served meals. Although the majority of the population adopted these recommendations during the COVID-19 pandemic, blue-collar workers, housewives, non-employed people and those with a low education were much less likely to do so. These subgroups may, therefore, be targeted for dining hygiene educational interventions in the future. 

Compared with the personal hygiene measures, the uptake of social distancing measures was much lower and decreased significantly over time. Behavioral health models such as the Health Belief Model posit that perceived susceptibility and perceived severity of disease are directly associated with likelihood of adopting a health behavior. Although nearly all respondents sought information about COVID-19 from various news sources, the higher incidence of disease and death in the third wave did not result in the sustained adoption of social distancing measures. It is also likely that lower adherence to social distancing measures during the third wave reflects the population’s fatigue with stringent measures such as the closure of bars, prohibition of restaurant dining in the evening hours and inability to travel outside of Hong Kong. However, we cannot rule out the possibility of lowered risk perception due to the population learning more about the disease [42]. Furthermore, when employees resumed working in offices, they were often urged through public reminders to observe personal hygiene measures, but public service messages did not discourage after-work gatherings. Difficulties in maintaining social distancing for workers and the lack of guidelines for workspaces may need to be addressed in a protracted pandemic. These findings indicate that in Hong Kong, despite strong adherence to mask use and hand hygiene practices, adherence to social distancing measures will be a challenge for long-term pandemic control. To improve Health-EDRM protection in urban contexts, periodic reassessment of population behaviors should be conducted by policy makers during prolonged biological emergencies.

### Research Limitations

This study has several limitations. Firstly, although the land-based telephone list covered around 85% of land-based telephones in Hong Kong during the two study periods [51], the households that were not on the list of land-based telephone service (institutionalized individuals, dormitory residents and individuals who live in boats and non-conventional dwellings) were not included. Secondly, our study sample was slightly older than the general population of Hong Kong but was comparable with the latest population census in terms of gender and area of residence. The third-wave sample comprised slightly older and lower-SES participants than the first wave. Hence, there may be some confounding by these characteristics in reporting the prevalence of preventive behaviors. Thirdly, this study was based upon self-reported data that may be subject to reporting bias. However, since the participants of the survey were anonymous, any reporting biases should be moderate. Lastly, changes in preventive behaviors between the serial cross-sectional studies cannot be conclusively stated to be related to the time period of the study, due to possible residual confounding by sociodemographic and unmeasured lifestyle variables. A longitudinal cohort-based study would be more appropriate to examine long-term trends in behavior changes.

## 5. Conclusions

In examining pattern changes of information sources, attitude and practice of COVID-19 protective measures, our results showed that the general population rapidly adopted COVID-19 preventive behaviors and that personal hygiene preventive measures remained high during the first nine months of the pandemic in Hong Kong. Mask wearing and continued hand hygiene are not behaviors requiring concerted public health efforts in Hong Kong. Despite the rising caseloads and deaths, social distancing behaviors waned significantly over time. Pandemic fatigue may, therefore, present a larger challenge for governments facing protracted pandemics.

Hygiene and social distancing messages should be targeted not only at high-risk sociodemographic groups but also at those showing greater pandemic fatigue. Health education programs aiming at improving COVID-19 knowledge may be helpful to improve continued adherence by those targeted groups.

## Figures and Tables

**Table 1:** Background characteristics of study sample and general pandemic perceptions.

	First Wave Sample (*n* = 765) March 2020	Third Wave Sample (*n* = 651) December 2020	*p*-Value (First Wave vs. Third Wave) ^b^	Hong Kong Census	Cohen’s w (First Wave vs. Hong Kong Census) ^b^
Gender			0.521		<0.001
Male	46.5% (356)	48.4% (315)		45.1%	
Female	53.5% (409)	51.6% (336)		54.9%	
Age			0.003		<0.001
18–24	9.3% (71)	6.9% (45)		9.5% ^c^	
25–44	32.4% (248)	26.3% (171)		35.3%	
45–64	39.6% (303)	41.8% (272)		36.8%	
65 or older	18.7% (143)	25.0% (163)		18.4%	
Marital status			0.406		<0.001
Non-married	39.8% (304)	37.5% (244)		39.9%	
Married	60.2% (459)	62.5% (406)		60.1%	
Residential district ^a^			0.660		<0.001
Hong Kong Island	19.2% (147)	17.5% (114)		17.2%	
Kowloon	30.2% (213)	29.8% (194)		30.6%	
New Territory	50.6% (387)	52.6% (342)		52.2%	
Education ^a,^*			0.007		0.005
Primary level or below	8% (61)	12.8% (83)		25.7%	
Secondary	43.3% (330)	43.7% (283)		43.7%	
Tertiary level	48.7% (371)	43.5% (282)		30.6%	
Household Income ^c^			0.012		0.007
<2000–7999	9.3% (66)	13.7% (86) ^d^		15.1%	
8000–19,999	14.1% (101)	16.2% (102)		25.9%	
20,000–39,999	26.6% (191)	27.3% (172)		27.8%	
40,000 or more	50.2% (360)	42.8% (269)		31.2%	
Employment status			0.003		0.005
White-collar worker	45.2% (341)	42.4% (275)		26.5%	
Blue-collar worker	17.0% (128)	14.3% (93)		24.7%	
Housewife	12.3% (93)	12.9% (84)		7.4%	
Full-time student	6.2% (47)	3.5% (23)		15.0%	
Unemployed/Retired	19.2% (145)	26.8% (174)		26.4%	

^a^ The Hong Kong Population Census data additionally included 15 to 17 years olds. ^b^ Samples from the first wave and third wave were compared using chi-square test, while the sample from the first wave and Hong Kong census were compared using Cohen’s effect size (small: 0.1; medium: 0.3; large: 0.5). ^c^ The analysis was conducted with household data.

**Table 2:** Preventive behaviors against COVID-19 *.

	1st Wave (*n* = 765) % (*n*)	3rd Wave (*n* = 651) % (*n*)	% Change	*p*-Value ^a^
**Hygiene practices**				
Wearing face mask outside the home	97.4% (745)	100.0% (651)	+2.6%	<0.001
Washing hands with soap	92.3% (706)	90.0% (586)	−2.3%	0.158
Use of serving utensils	74.2% (568)	69.4% (452)	−4.8%	0.051
Bring own utensils when dining out ^†^	7.9% (52)	5.3% (31)	−2.6%	0.086
**Social distancing practices**				
Avoidance of international travel to high-risk regions	85.8% (656)	76.6% (498)	−9.1%	<0.001
Avoidance of social gatherings	80.5% (616)	72.0% (469)	−8.5%	<0.001
Avoidance of public places and public transport	53.3% (408)	26.0% (169)	−27.5%	<0.001
Avoidance of dine-in services at restaurants by using takeout/home delivery services	34.4% (262)	44.8% (290)	+10.4%	<0.001

* % of respondents who stated that they “Always” or “Usually” engaged in the behaviors. ^a^ Two-proportions z-test with continuity correction was used to test the difference between the two waves of studies. ^†^ This analysis only included people who will go outdoors for a meal during the epidemic.

**Table 3:** Factors associated with compliance with personal hygiene and social distancing measures in Hong Kong COVID-19 1st wave and 3rd wave.

	PERSONAL HYGIENE MEASURES			SOCIAL DISTANCING MEASURES			
	Hand Hygiene with Soap and Alcohol	Strict Use of Serving Utensils for Shared Dishes	Bringing Own Eating Utensils When Dining out	Avoidance of Social Gatherings	Order Takeout/Food Delivery	Avoidance of Public Places/Public Transport	Avoidance of International Travel
	AOR (95% CI)	AOR (95% CI)	AOR (95% CI)	AOR (95% CI)	AOR (95% CI)	AOR (95% CI)	AOR (95% CI)
**Time period**							
Wave 1	1.00	1.00	1.00	1.00	1.00	1.00	1.00
Wave 3	0.88 (0.60–1.30)	0.75 (0.59–0.95) *	0.63 (0.42–1.05)	**0.62 (0.48–0.80) ^§^**	**1.83 (1.45–2.31) ^§^**	**0.27 (0.22–0.35) ^§^**	**0.53 (0.40–0.70) ^§^**
**Age**							
65+	1.00	1.00	1.00	1.00	1.00	1.00	1.00
50–64	1.61 (1.01–2.57) *	0.80 (0.54–1.20)	0.45 (0.22–0.92) *	1.01 (0.65–1.56)	1.37 (0.93–2.00)	0.93 (0.64–1.37)	0.82 (0.51–1.31)
35–49	**3.03 (1.62–5.69) ^†^**	0.68 (0.43–1.08)	1.43 (0.79–2.62)	1.07 (0.65–1.77)	**2.30 (1.53–3.46) ^§^**	1.21 (0.78–1.87)	1.00 (0.58–1.71)
18–34	**3.19 (1.64–6.19) ^†^**	**0.41 (0.25–0.67) ^§^**	0.67 (0.33–1.35)	0.86 (0.51–1.47)	**2.60 (1.71–3.96) ^§^**	0.85 (0.53–1.36)	0.84 (0.48–1.47)
**Gender**							
Male	1.00	1.00	1.00	1.00	1.00	1.00	1.00
Female	**2.08 (1.40–3.09) ^§^**	1.37 (1.06–1.78) *	1.57 (0.98–2.49)	**1.44 (1.10–1.90) ^†^**	**0.71 (0.56–0.89) ^†^**	1.04 (0.81–1.35)	1.23 (0.92–1.65)
**Education**							
Primary	1.00	1.00	1.00	1.00	1.00	1.00	1.00
Secondary	1.85 (1.07–3.20) *	1.54 (1.01–2.35) *	0.73 (0.32–1.66)	**2.00 (1.29–3.11) ^†^**	1.10 (0.68–1.78)	1.61 (1.04–2.48) *	1.01 (0.58–1.73)
Post-secondary	2.03 (1.12–3.69) *	**1.88 (1.19–2.99) ^†^**	0.72 (0.30–1.76)	**2.65 (1.64–4.30) ^§^**	1.00 (0.60–1.66)	**2.44 (1.53–3.89) ^§^**	1.07 (0.59–1.93)
**Household income**							
<2000–7999 HKD	1.00	1.00	1.00	1.00	1.00	1.00	1.00
8000–19,999 HKD	0.92 (0.48–1.78)	1.20 (0.73–1.98)	0.68 (0.26–1.78)	1.20 (0.70–2.05)	1.25 (0.74–2.11)	0.91 (0.56–1.49)	0.93 (0.52–1.66)
20,000–39,999 HKD	1.00 (0.52–1.94)	1.20 (0.74–1.96)	0.77 (0.30–1.99)	1.02 (0.60–1.72)	1.50 (0.91–2.47)	0.73 (0.45–1.18)	1.25 (0.70–2.23)
40,000+ HKD	0.90 (0.44–1.83)	1.26 (0.76–2.09)	0.81 (0.31–2.14)	1.28 (0.74–2.20)	**2.45 (1.49–4.06) ^§^**	1.17 (0.72–1.92)	1.18 (0.66–2.12)
**Occupation**							
White collar	1.00	1.00	1.00	1.00	1.00	1.00	1.00
Blue collar	0.65 (0.35–1.20)	**0.50 (0.35–0.73) ^§^**	1.29 (0.68–2.44)	1.27 (0.86–1.89)	0.79 (0.56–1.11)	0.77 (0.53–1.12)	0.91 (0.62–1.33)
Housewife	0.77 (0.35–1.72)	0.61 (0.39–0.97) *	0.88 (0.39–2.08)	1.63 (0.98–2.70)	0.65 (0.41–1.03)	**3.53 (2.32–5.38) ^§^**	1.85 (1.09–3.16) *
Students	1.70 (0.36–8.10)	0.65 (0.37–1.17)	0.21 (0.03–1.69)	0.93 (0.50–1.75)	0.92 (0.52–1.61)	1.92 (1.06–3.47) *	1.33 (0.64–2.76)
Non-employed	0.65 (0.35–1.22)	0.66 (0.44–1.00) *	1.08 (0.47–2.46)	1.69 (1.08–2.65) *	0.84 (0.56–1.26)	**2.24 (1.52–3.31) ^§^**	1.25 (0.78–1.99)
**Has chronic disease**							
No	1.00	1.00	1.00	1.00	1.00	1.00	1.00
Yes	1.05 (0.67–1.66)	0.93 (0.68–1.27)	0.68 (0.34–1.35)	1.09 (0.78–1.53)	1.09 (0.79–1.50)	0.96 (0.70–1.32)	0.87 (0.61–1.25)

Note: * indicates *p* < 0.05; ^†^ indicates *p* < 0.01; ^§^ indicates *p* < 0.001. *p*-values with bold indicate statistically significant differences between the groups. For all multivariable models, time period and age variables were forced into the final model. For all other variables, odds ratios and 95% CI are shown for non-significant variables prior to being dropped from the final multivariable model.

## Data Availability

The datasets generated during the current study are available from the corresponding author on reasonable request.

## Appendix A

**Table A1:** Factors associated with compliance with personal hygiene measures in Hong Kong COVID-19 first wave and third wave.

	Mask Wearing 1st Wave		Mask Wearing 3rd Wave		Increased Hand Hygiene * 1st Wave		Increased Hand Hygiene * 3rd Wave		Use Serving Utensil 1st Wave		Use Serving Utensil 3rd Wave	
	%	AOR (95% CI)	%		%	AOR (95% CI)	%	AOR (95% CI)	%	AOR (95% CI)	%	AOR (95% CI)
**Gender**												
Male	94.9%	1.00	100.0%	NA	89.6%	1.00	86.7%	1.00	70.8%	1.00	67.3%	1.00
Female	99.5%	11.14 (2.55–48.64) ^†^	100.0%	NA	94.6%	2.17 (1.22–3.87) ^†^	93.2%	2.16 (1.13–4.10) *	77.3%	1.42 (0.98–2.07)	71.4%	1.28 (0.87–1.89)
**Education**												
Primary	93.4%	1.00	100.0%	NA	80.3%	1.00	81.9%	1.00	73.8%	1.00	57.8%	1.00
Secondary	97.3%	1.80 (0.37–8.70)	100.0%	NA	91.8%	1.89 (0.84–4.25)	91.2%	2.01 (0.93–4.33)	70.6%	0.97 (0.48–1.98)	70.0%	2.15 (1.23–3.77) ^†^
Post-secondary	98.1%	1.26 (0.23–7.00)	100.0%	NA	94.9%	2.51 (1.03–6.15) *	91.5%	1.49 (0.62–3.56)	77.9%	1.35 (0.62–2.91)	72.3%	2.07 (1.11–3.85) *
**Age**				NA								
65+	93.7%	1.00	100.0%	NA	81.8%	1.00	85.3%	1.00	66.5%	1.00	72.4%	1.00
50–64	97.8%	3.35 (1.08–10.38) *	100.0%	NA	92.1%	2.42 (1.22–4.80) *	88.5%	0.93 (0.42–2.04)	77.0%	1.03 (0.58–1.83)	69.9%	0.66 (0.38–1.15)
35–49	98.0%	3.06 (0.91–10.33)	100.0%	NA	96.6%	4.66 (1.87–11.6) ^†^	93.6%	1.33 (0.46–3.85)	78.0%	0.83 (0.44–1.57)	73.0%	0.62 (0.32–1.19)
18–34	99.0%	6.66 (1.40–31.75) *	100.0%	NA	95.8%	3.87 (1.59–9.41) ^†^	95.0%	1.65 (0.47–5.77)	74.8%	0.60 (0.31–1.14)	60.3%	0.35 (0.17–0.71) ^†^
**Household income**				NA								
<2000–7999 HKD	93.9%	1.00	100.0%	NA	81.8%	1.00	84.9%	1.00	74.2%	1.00	64.0%	1.00
8000–19,999 HKD	96.0%	1.97 (0.38–10.16)	100.0%	NA	90.1%	1.27 (0.47–3.44)	86.3%	0.76 (0.31–1.84)	70.3%	0.91 (0.43–1.93)	71.6%	1.61 (0.82–3.16)
20,000–39,999 HKD	97.4%	2.11 (0.39–11.40)	100.0%	NA	92.7%	0.87 (0.32–2.41)	91.3%	1.09 (0.45–2.66)	72.8%	0.99 (0.46–2.11)	68.6%	1.40 (0.73–2.68)
40,000+ HKD	98.6%	3.53 (0.52–23.98)	100.0%	NA	94.4%	0.83 (0.28–2.43)	91.8%	0.93 (0.36–2.41)	76.4%	1.02 (0.47–2.20)	71.0%	1.51 (0.77–2.99)
**Occupation**				NA								
White collar	98.5%	1.00	100.0%	NA	95.0%	1.00	94.2%	1.00	79.2%	1.00	75.3%	1.00
Blue collar	96.1%	0.53 (0.11–2.61)	100.0%	NA	91.4%	0.75 (0.30–1.96)	87.1%	0.49 (0.20–1.22)	65.6%	0.49 (0.31–0.77) ^†^	55.9%	0.39 (0.23–0.68) ^†^
Housewife	97.8%	0.10 (0.00–2.96)	100.0%	NA	93.5%	1.01 (0.29–3.48)	90.5%	0.46 (0.15–1.40)	77.4%	0.77 (0.42–1.39)	66.7%	0.58 (0.31–1.08)
Students	97.9%	0.29 (0.01–6.00)	100.0%	NA	97.9%	2.09 (0.23–19.29)	95.7%	1.09 (0.11–10.48)	61.7%	0.57 (0.28–1.16)	52.2%	0.55 (0.21–1.44)
Non-employed	95.2%	2.75 (0.38–19.87)	100.0%	NA	84.1%	0.78 (0.29–2.12)	84.5%	0.48 (0.20–1.17)	73.1%	0.59 (0.33–1.07)	70.7%	0.61 (0.35–1.08)
**Chronic Disease**				NA								
No	97.4%	1.00	100.0%	NA	92.9%	1.00	91.1%	1.00	74.4%	1.00	69.4%	1.00
Yes	97.2%	1.64 (0.46–5.90)	100.0%	NA	89.4%	1.26 (0.62–2.59)	86.6%	1.02 (0.54–1.92)	73.8%	0.96 (0.59–1.56)	70.1%	0.97 (0.62–1.53)

Note: * indicates *p* < 0.05; ^†^ indicates *p* < 0.01; ^§^ indicates *p* < 0.001.

**Table A2:** Factors associated with compliance with social distancing measures in Hong Kong COVID-19 first wave and third wave.

	**Avoidance of Social Gatherings 1st Wave**		**Avoidance of Social Gatherings** **3rd Wave**		**Order Takeaway** **More Often 1st Wave**		**Order Takeaway** **More Often in 3rd Wave**	
	**%**	**AOR** **(95% CI)**	**%**		**%**	**AOR** **(95% CI)**	**%**	**AOR** **(95% CI)**
**Gender**								
Male	75.6%	1.00	70.8%	1.00	36.1%	1.00	51.0%	1.00
Female	84.8%	1.84 (1.22–2.77) ^†^	73.2%	1.24 (0.84–1.82)	32.9%	0.95 (0.68–1.34)	38.9%	0.57 (0.39–0.83) ^†^
**Education**								
Primary	75.4%	1.00	60.2%	1.00	23.3%	1.00	22.0%	1.00
Secondary	78.5%	1.30 (0.60–2.83)	74.2%	1.94 (1.11–3.39) *	31.8%	0.75 (0.36–1.54)	42.2%	1.43 (0.75–2.70)
Post-secondary	83.0%	1.97 (0.85–4.58)	73.0%	2.01 (1.11–3.65) *	38.8%	0.73 (0.34–1.58)	54.1%	1.21 (0.60–2.42)
Age								
65+	78.3%	1.00	75.5%	1.00	20.0%	1.00	25.8%	1.00
50–64	80.2%	1.20 (0.59–2.46)	73.0%	0.82 (0.43–1.54)	31.7%	1.46 (0.76–2.80)	38.6%	1.32 (0.75–2.34)
35–49	82.8%	1.24 (0.56–2.75)	72.3%	0.81 (0.38–1.72)	38.7%	1.88 (0.92–3.86)	60.3%	2.68 (1.39–5.19) ^†^
18–34	80.1%	1.25 (0.53–2.96)	65.3%	0.56 (0.25–1.25)	43.5%	2.28 (1.07–4.86) *	63.6%	2.71 (1.32–5.56) ^†^
**Household income**								
<2000–7999	84.8%	1.00	68.6%	1.00	19.0%	1.00	22.4%	1.00
8000–19,999	76.2%	0.60 (0.25–1.44)	79.4%	1.62 (0.82–3.19)	26.7%	1.17 (0.52–2.63)	30.4%	1.27 (0.63–2.58)
20,000–39,999	78.0%	0.68 (0.28–1.67)	69.2%	0.86 (0.48–1.56)	30.9%	1.19 (0.53–2.67)	42.7%	1.69 (0.87–3.30)
40,000 or more	82.5%	0.89 (0.36–2.22)	72.1%	0.93 (0.51–1.68)	42.8%	1.85 (0.82–4.15)	60.2%	2.66 (1.35–5.27) ^†^
**Occupation**								
White collar	79.8%	1.00	70.2%	1.00	39.6%	1.00	59.0%	1.00
Blue collar	81.3%	1.54 (0.85–2.79)	68.8%	1.00 (0.56–1.78)	39.1%	1.12 (0.70–1.81)	33.3%	0.46 (0.27–0.79) ^†^
Housewife	87.1%	2.33 (0.99–5.53)	73.8%	1.49 (0.73–3.06)	20.7%	0.58 (0.30–1.10)	27.7%	0.70 (0.37–1.33)
Students	74.5%	0.82 (0.34–1.94)	60.9%	0.97 (0.36–2.61)	40.4%	0.90 (0.43–1.87)	69.6%	1.00 (0.37–2.74)
Non-employed	79.3%	1.40 (0.69–2.88)	77.0%	1.81 (0.95–3.42)	24.5%	0.80 (0.44–1.48)	33.3%	0.80 (0.46–1.40)
**Chronic Disease**								
No	80.6%	1.00	71.0%	1.00	35.5%	1.00	47.8%	1.00
Yes	80.1%	1.17 (0.68–2.01)	75.8%	1.14 (0.71–1.82)	29.3%	1.18 (0.74–1.89)	35.5%	1.18 (0.74–1.89)
	**Avoid Going to Public Places or Using Public Transport** **in 1st Wave**		**Avoid Going to Public Places or** **Using Public Transport** **in 3rd Wave**		**Avoidance of Regions Outside Hong Kong in 1st Wave**		**Avoidance of Outside Hong Kong** **in 3rd Wave**	
	**%**	**AOR** **(95% CI)**	**%**		**%**	**AOR** **(95% CI)**	**%**	**AOR** **(95% CI)**
**Gender**								
Male	49.9%	1.00	24.8%	1.00	82.9%	1.00	75.2%	1.00
Female	56.6%	1.03 (0.74–1.43)	27.1%	1.07 (0.71–1.63)	88.3%	1.37 (0.87–2.15)	77.9%	1.03 (0.69–1.55)
**Education**								
Primary	50.8%	1.00	22.9%	1.00	88.5%	1.00	79.5%	1.00
Secondary	50.2%	1.38 (0.72–2.64)	25.4%	2.01 (1.08–3.75) *	86.7%	1.01 (0.40–2.55)	76.0%	1.01 (0.52–1.98)
Post-secondary	57.0%	1.70 (0.85–3.41)	27.0%	3.14 (1.59–6.22) ^†^	84.4%	0.92 (0.35–2.47)	76.2%	1.17 (0.55–2.47)
**Age**								
65+	52.4%	1.00	36.2%	1.00	87.4%	1.00	82.1%	1.00
50–64	57.9%	1.29 (0.72–2.64)	25.2%	0.75 (0.43–1.29)	83.3%	0.88 (0.39–1.97)	75.7%	0.79 (0.41–1.52)
35–49	48.5%	2.29 (1.19–4.43)	23.4%	0.69 (0.36–1.35)	88.7%	1.51 (0.60–3.83)	74.5%	0.67 (0.31–1.44)
18–34	56.6%	1.95 (0.96–3.94)	16.5%	0.38 (0.17–0.84) *	84.3%	0.94 (0.36–2.46)	73.6%	0.63 (0.28–1.46)
**Household income**								
<2000–7999	59.1%	1.00	31.4%	1.00	86.4%	1.00	80.0%	1.00
8000–19,999	52.5%	0.82 (0.41–1.62)	25.5%	1.02 (0.51–2.04)	85.1%	1.01 (0.39–2.60)	74.5%	0.93 (0.44–1.98)
20,000-39,999	44.0%	0.56 (0.28–1.12)	22.1%	0.96 (0.49–1.87)	87.4%	1.40 (0.53–3.69)	77.3%	1.22 (0.58–2.56)
40,000 or more	56.7%	0.98 (0.49–1.98)	26.4%	1.44 (0.72–2.90)	84.7%	1.23 (0.47–3.26)	75.5%	1.23 (0.57–2.64)
**Occupation**								
White collar	51.3%	1.00	20.4%	1.00	84.5%	1.00	74.2%	1.00
Blue collar	38.3%	0.74 (0.46–1.19)	12.9%	0.71 (0.34–1.45)	83.6%	1.05 (0.56–1.96)	71.0%	0.94 (0.52–1.69)
Housewife	71.0%	3.53 (1.89–6.59) ^§^	41.7%	3.33 (1.78–6.25) ^§^	91.4%	2.04 (0.78–5.32)	84.3%	1.94 (0.88–4.27)
Students	59.6%	1.34 (0.64–2.78)	26.1%	2.76 (0.89–8.53)	89.4%	1.72 (0.58–5.08)	73.9%	1.14 (0.39–3.37)
Non-employed	60.7%	2.40 (1.34–4.31) ^†^	34.5%	2.00 (1.12–3.57) *	86.2%	1.55 (0.70–3.43)	80.5%	1.38 (0.72–2.64)
**Chronic Disease**								
No	52.7%	1.00	25.2%	1.00	85.9%	1.00	76.6%	1.00
Yes	56.7%	1.11 (0.72–1.72)	28.7%	0.78 (0.49–1.26)	85.1%	0.94 (0.52–1.72)	77.1%	0.84 (0.52–1.37)

Note: * indicates *p* < 0.05; ^†^ indicates *p* < 0.01; ^§^ indicates *p* < 0.001.
